# Supplementary material for: Biogeographical network analysis of plant species distribution in the Mediterranean region
Source: Ecol Evol. 2018 Dec 21;9(1):237–50. doi: 10.1002/ece3.4718 (PMC6342112; doi:10.1002/ece3.4718)
Supplement: Supplementary file 1 [file ECE3-9-237-s001.pdf]

Supplementary information for Biogeographical network analysis of plant species distribution in the Mediterranean region

Maxime Lenormand[1], Guillaume Papuga[2,3], Olivier Argagnon[2], Maxence Soubeyrand[1], Guilhem De Barros[2], Samuel Alleaume[1] and Sandra Luque[1]

[1] Irstea, UMR TETIS, 500 rue JF Breton, FR-34093 Montpellier, France
[2] Conservatoire botanique national méditerranéen de Porquerolles, Parc scientifique Agropolis, 2214 boulevard de la Lironde, 34980 Montferrier sur Lez, France
[3] UMR 5175 CEFE, CNRS, 1919 route de Mende, 34293 Montpellier cedex 5, France

Interactive web application

An interactive web application has been designed to provide an easy-to-use interface to visualize the results and the maps of the main paper and the Supplementary Information (Figure S1). The source code of the interactive web application[1] can be downloaded from[2].

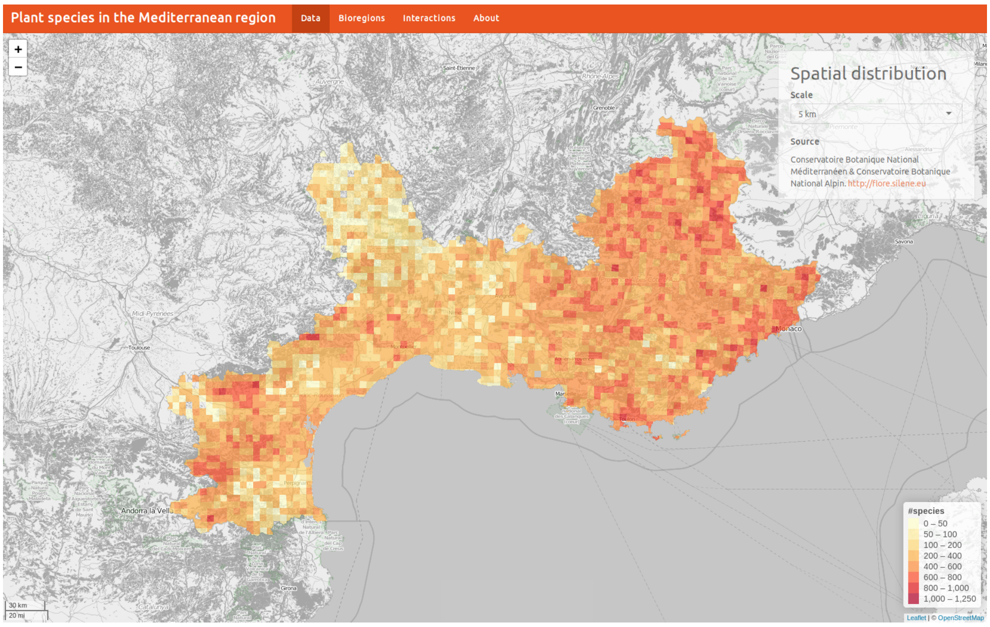

Figure S1: Screenshot of the interactive web application.

[1] https://maximelenormand.shinyapps.io/Biogeo/
[2] www.maximelenormand.com/Codes

Influence of scale on the biogeographical regions delineation

In order to assess the impact of the spatial resolution on the results, we also applied the analysis with a grid composed of squares of lateral size l = 10 km (Figure S2). The spatial coherence, defined as the ratio between the number of grid cells in the largest patch and the total number of grid cells [1], is displayed for both scale in Table S1.

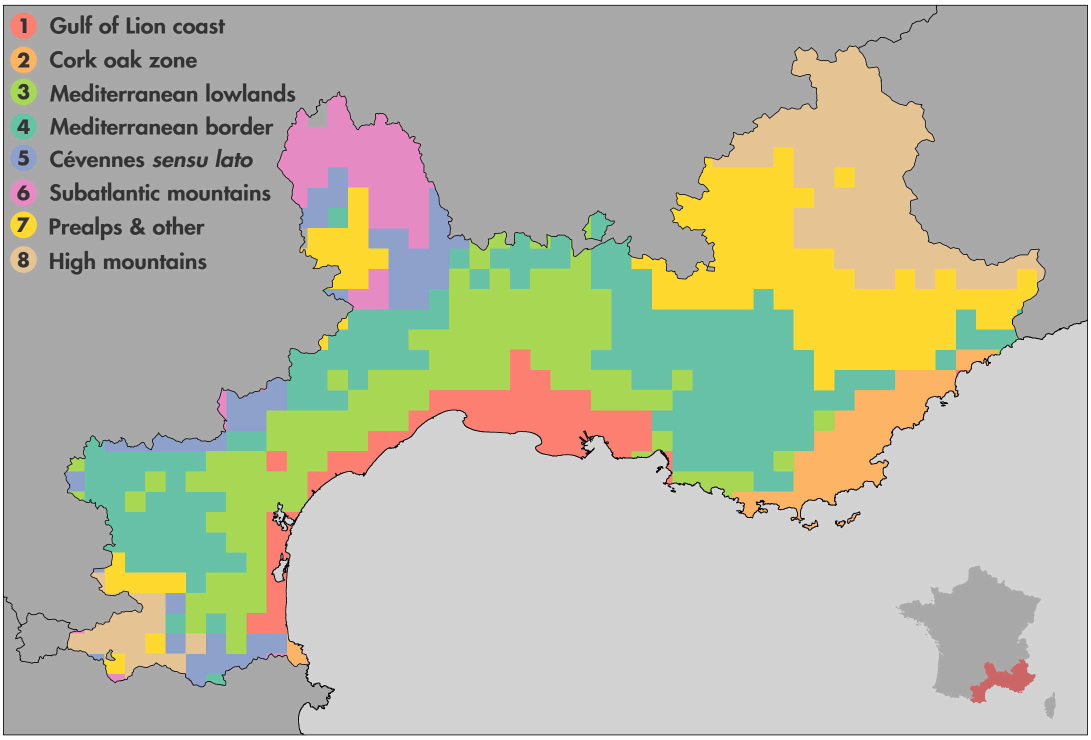

Figure S2: **Biogeographical regions based on similarity in plant species (l = 10 km).** Eight biogeographical regions have been identified. 1. Gulf of Lion coast in red. 2. Cork oak zone in orange. 3. Mediterranean lowlands in light green. 4. Mediterranean border in dark green. 5. Cévennes *sensu lato* in purple. 6. Subatlantic mountains in pink. 7. Prealps and other medium mountains in yellow. 8. High mountains in brown.

Table S1: **Spatial coherence of the biogeographical regions according to the scale.**

**Bioregion**	n_i **(l=5)**	SP_i **(l=5)**	n_i **(l=10)**	SP_i **(l=10)**
**1**	170	0.63	63	0.75
**2**	183	0.73	47	0.87
**3**	529	0.86	124	0.83
**4**	807	0.57	164	0.51
**5**	120	0.50	45	0.31
**6**	152	0.70	48	0.79
**7**	400	0.67	114	0.75
**8**	246	0.78	110	0.77

Comparison of OSLOM to standard clustering methods

In this work, bioregions are delineating using the community detection algorithm OSLOM applied on a weighted undirected spatial network whose intensity of links between grid cells are measured with the Jaccard similarity coefficient. This algorithm is nonparametric in the sense that it identifies statistically significant communities with respect to a global null model, and therefore the number of communities does not need to be defined *a priori*. In order to assess the accuracy of the method, we compared the results obtained with OSLOM with the ones obtained with standard hierarchical clustering methods. Not that these standard methods cannot be directly applied on the spatial network described above, we first need to transform the network into a dissimilarity matrix. Three different agglomeration methods have been tested: average (UPGMA), mcquitty (WPGMA) and Ward[1]. To choose the number of clusters, we used the average silhouette index $\bar{S}$ [2]. For each cell g, we can compute $a(g)$ the average dissimilarity of g (based on the Jaccard index in our case) with all the other cells in the cluster to which g belongs. In the same way, we can compute the average dissimilarities of g to the other clusters and define $b(g)$ as the lowest average dissimilarity among them. Using these two quantities, we compute the silhouette index $s(g)$ defined as,

$$s(g) = \frac{b(g) - a(g)}{max\{a(g), b(g)\}} \tag{1}$$

which measures how well clustered g is. This measure is comprised between -1 for a very poor clustering quality and 1 for an appropriately clustered g. We choose the number of clusters that maximize the average silhouette index over all the grid cells $\bar{S} = \sum_{g=1}^{n} s(g)/n$.

UPGMA and WPGMA failed to detect any coherent partitions, most of the grid cells were gathered in a giant cluster component even increasing significantly the number of clusters. Better results were obtained with Ward's method. The average Silhouette index as a function of the number of clusterS is shown in Figure S3.

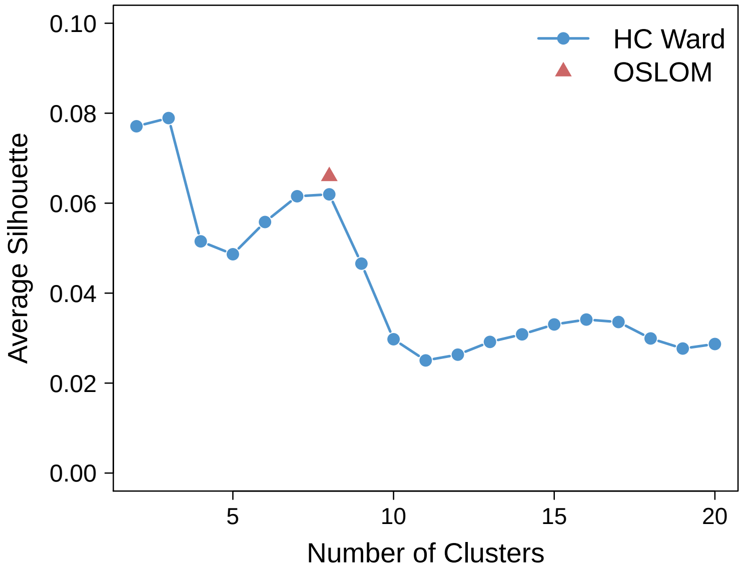

Figure S3: **Average Silhouette as a function of the number of clusters obtained with Ward's clustering (in blue) and OSLOM (in red).**

[1] method="average", "mcquitty" and "ward.D2" with the hclust R function

Two optimal partitions have been detected with the average Silhouette index. It is interesting to note that the number of clusters of the second partition is the same that the one automatically detected with OSLOM (Figure S3).

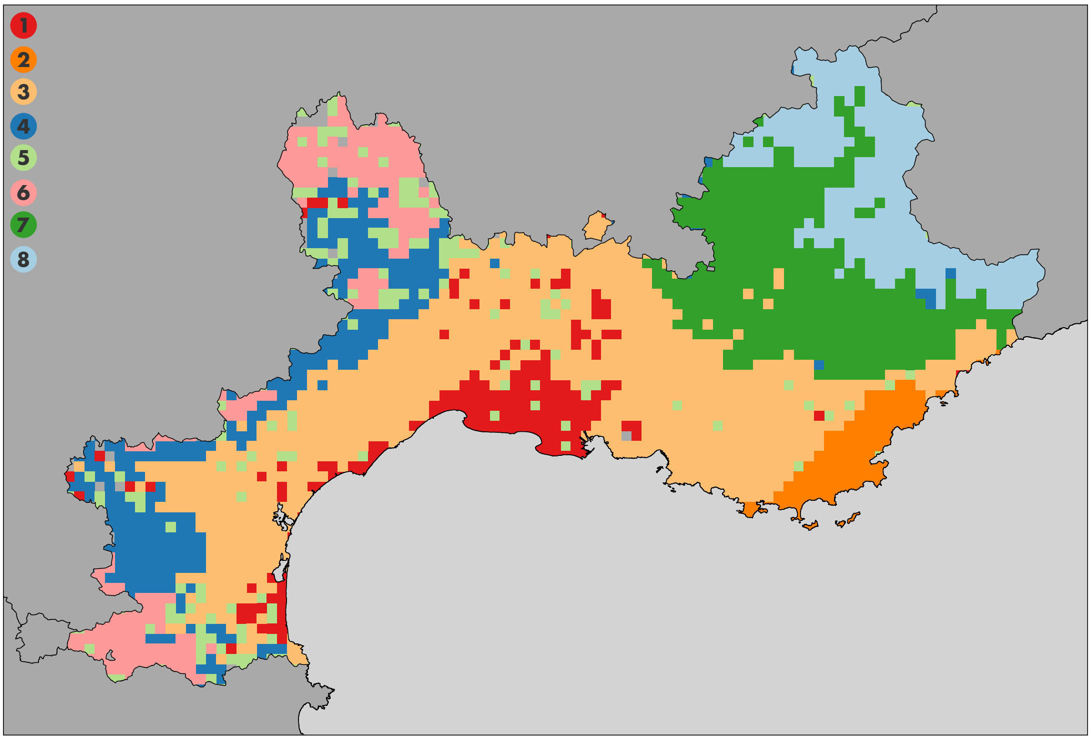

Figure S4: **Biogeographical regions based on similarity in plant species obtained with Ward's clustering (l = 5 km).** Eight biogeographical regions have been identified.

A map of the eight optimal bioregions obtained with Ward's method is display in Figure S4. In order to compare the two partitions a contingency between the partitions obtained with Ward's method and OSLOM is shown in Table S2.

Table S2: **Contingency tables between the partitions obtained with Ward (in row) and OSLOM (in column).**

**Bioregion**	**1**	**2**	**3**	**4**	**5**	**6**	**7**	**8**
**1**	**125**	2	54	0	4	0	0	0
**2**	0	**115**	0	2	0	0	0	0
**3**	28	47	**435**	396	0	0	0	0
**4**	0	4	4	**187**	48	1	43	1
**5**	17	15	34	20	**53**	32	8	15
**6**	0	0	0	0	15	**119**	38	33
**7**	0	0	0	200	0	0	**216**	0
**8**	0	0	0	2	0	0	95	**197**

Comparison with other delineations

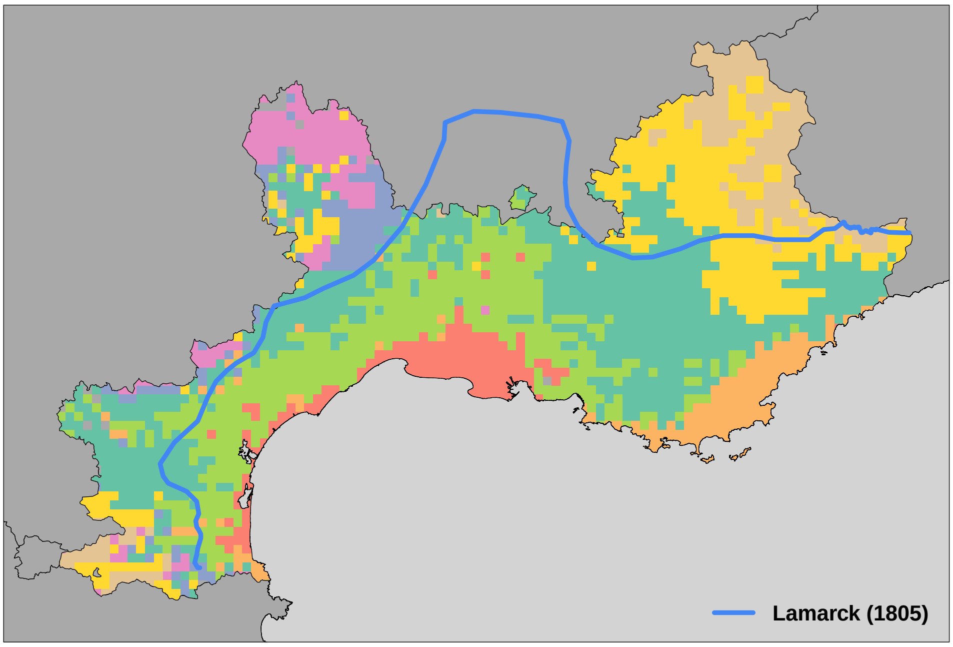

Figure S5: **Comparison of the results obtained with OSLOM (l = 5 km) with Lamarck's limit of the Mediterranean level [3, 4].**

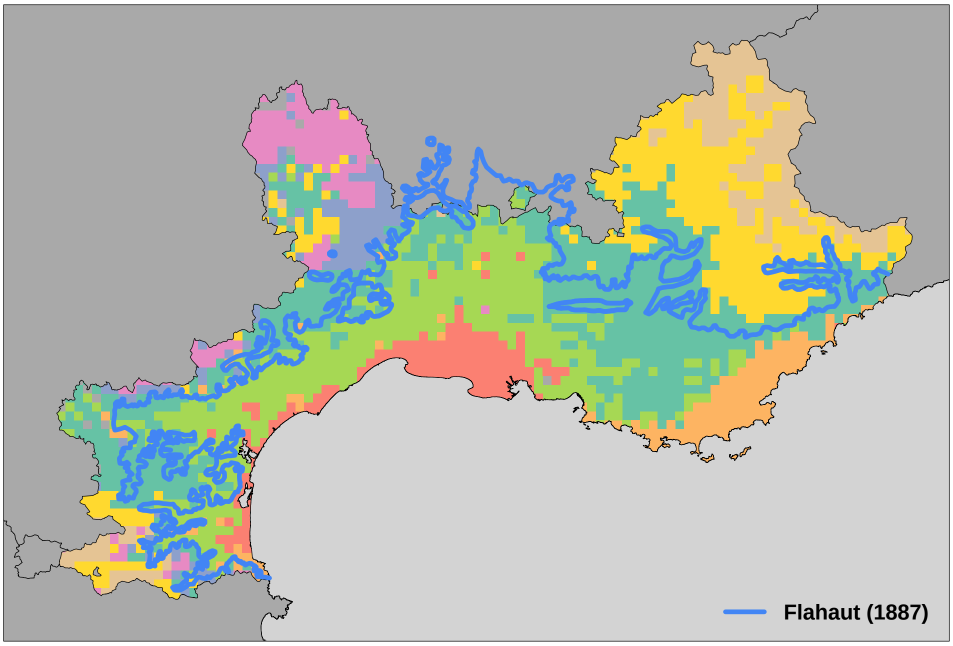

Figure S6: **Comparison of the results obtained with OSLOM (l = 5 km) with Flahaut limit of the olive tree distribution [5].**

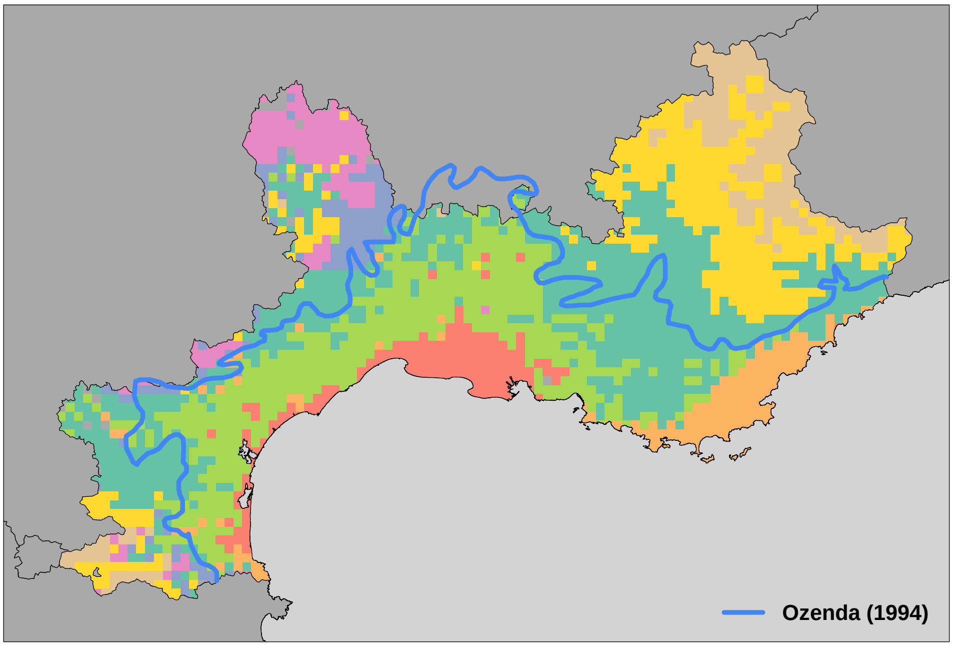

Figure S7: **Comparison of the results obtained with OSLOM (l = 5 km) with Ozenda's mediterranean/supramediterranean limit [6].**

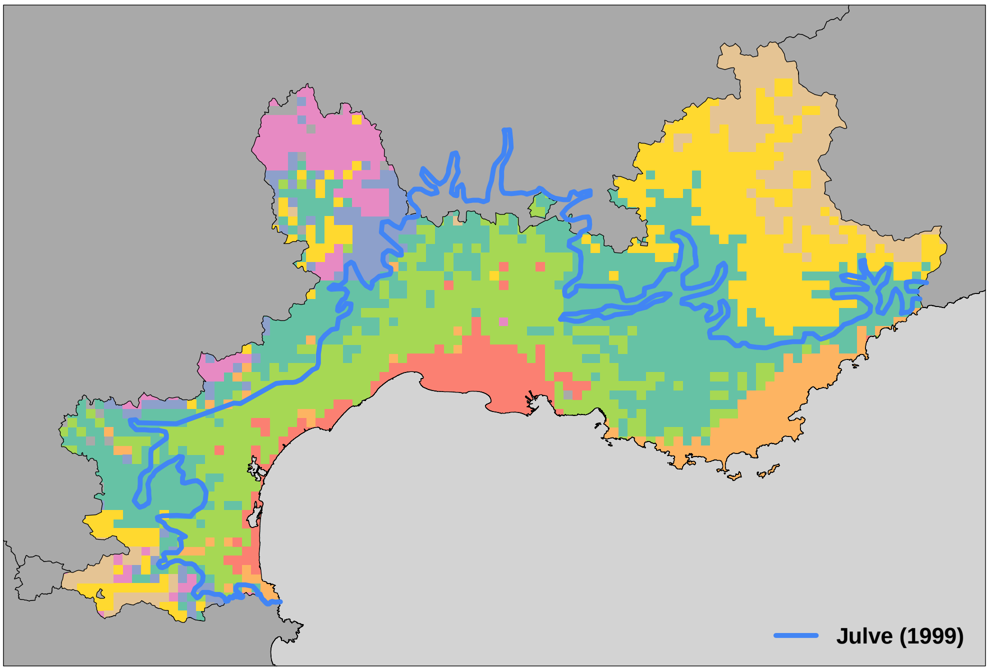

Figure S8: **Comparison of the results obtained with OSLOM (l = 5 km) with Julve's mediterranean/supramediterranean limit [7].**

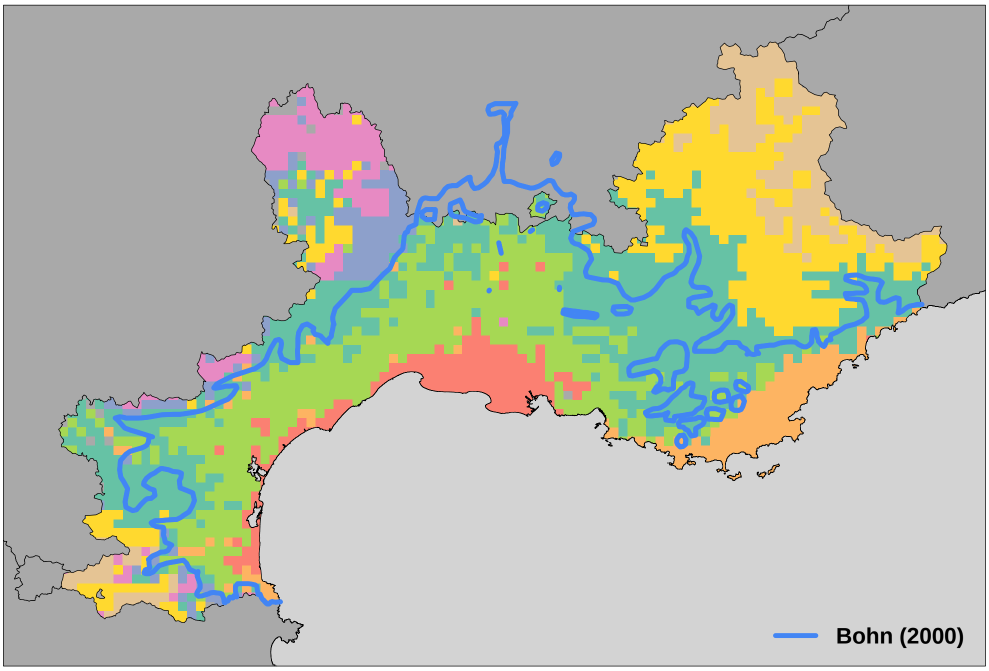

Figure S9: **Comparison of the results obtained with OSLOM (l = 5 km) with Bohn's mediterranean/supramediterranean limit [8].**

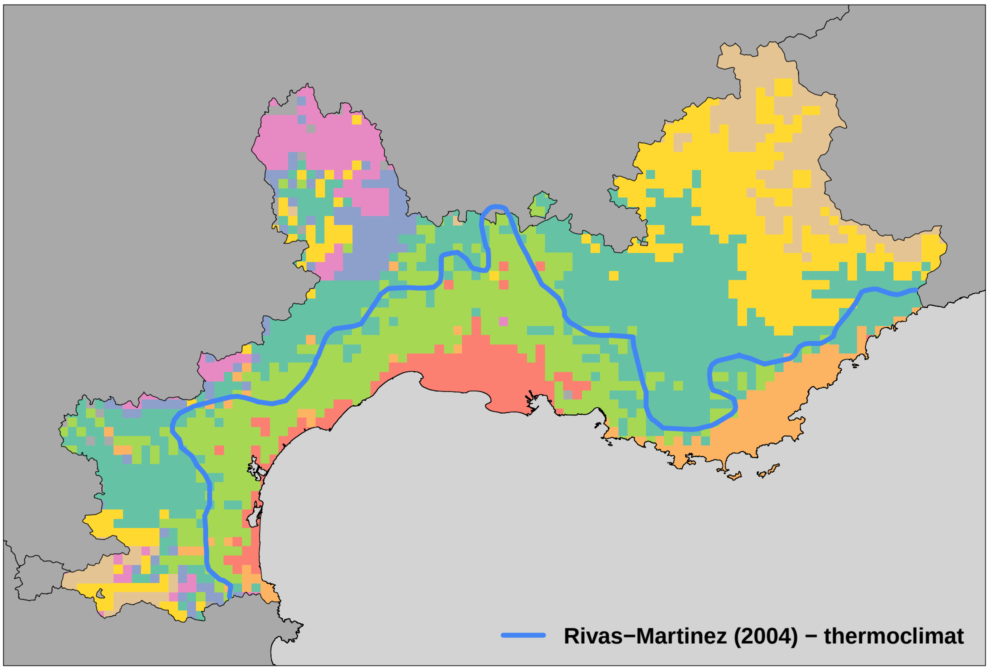

Figure S10: **Comparison of the results obtained with OSLOM (l = 5 km) with Rivas-Martínez's thermoclimatic limit [9].**

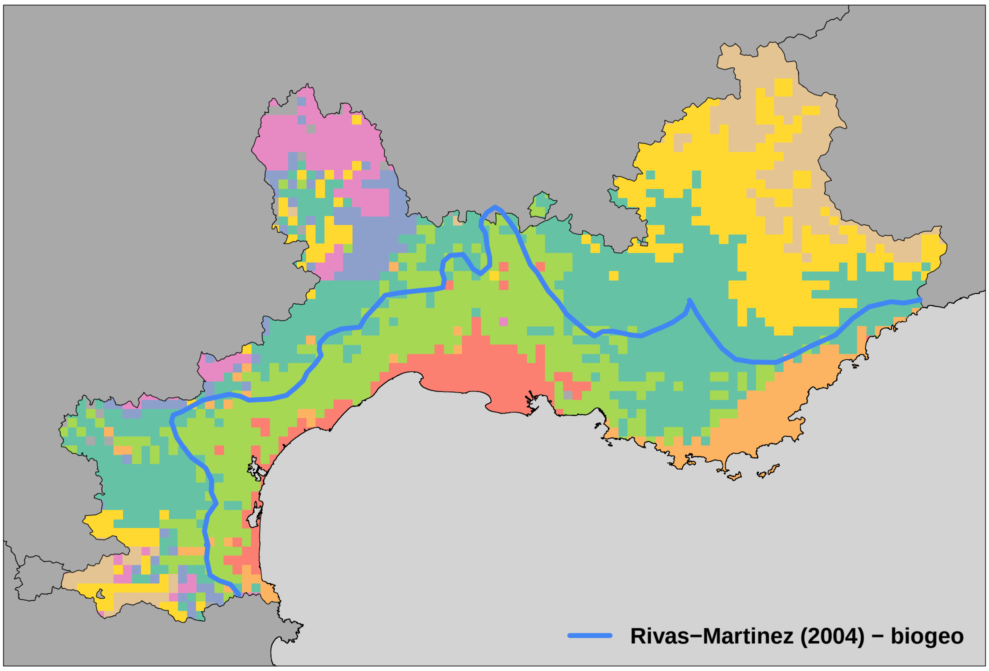

Figure S11: **Comparison of the results obtained with OSLOM (l = 5 km) with Rivas-Martínez's biogeographical limit [10].**

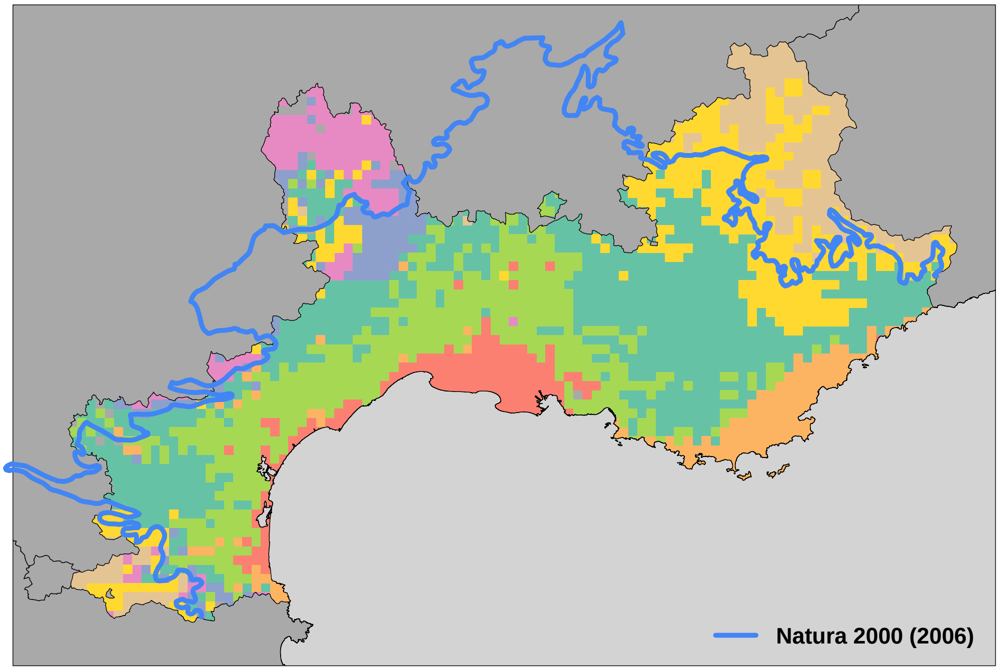

Figure S12: **Comparison of the results obtained with OSLOM (l = 5 km) with the Natura 2000's limit [11].**

Supplementary Figures

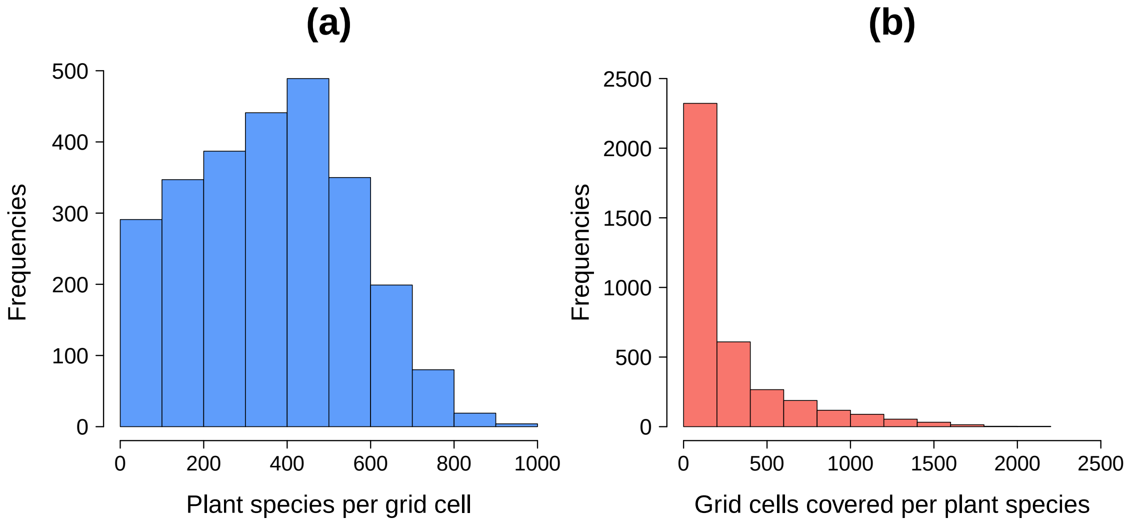

Figure S13: Histograms of the degree distributions of the biogeographical bipartite network. Histogram of the number of plant species per grid cell (a) and the number of cells covered per plant species (b).

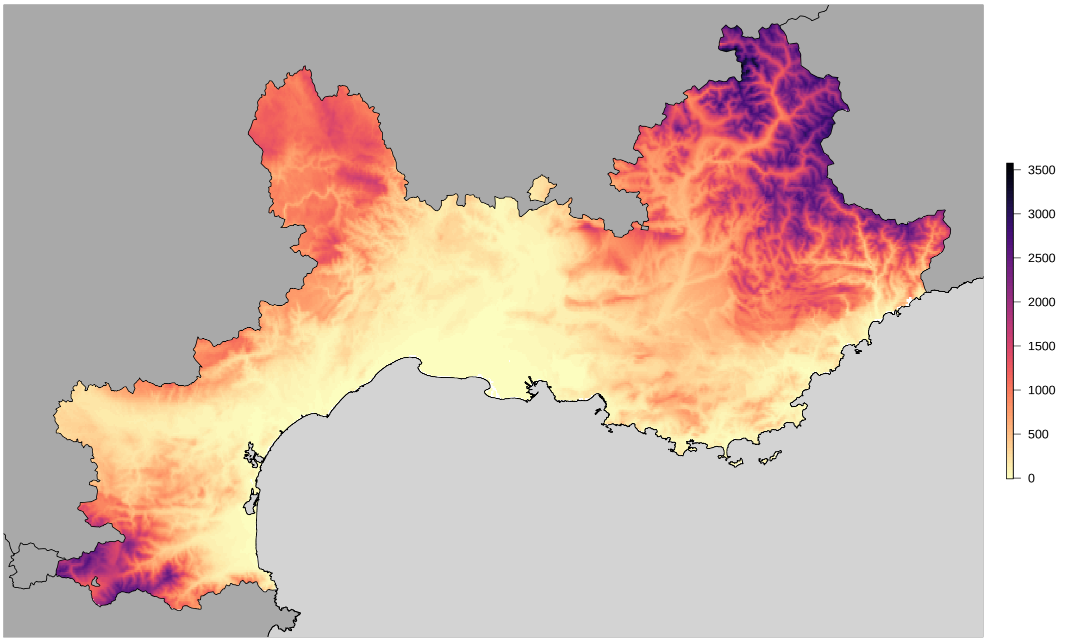

Figure S14: Altitude map of the studied area (in meters).

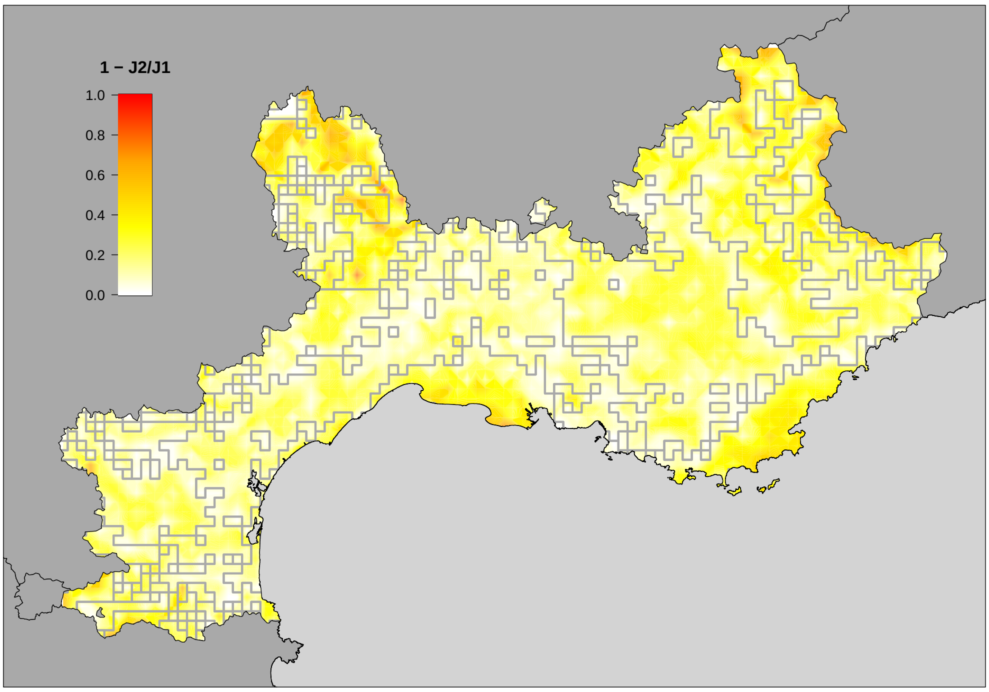

Figure S15: **Uncertainty map (l = 5 km).** For a given cell, J1 represents the average Jaccard similarity index between this cell and all the cells that belong to its cluster, and J2 represents the average Jaccard similarity index between this cell and all the cells belonging to the second closest cluster (based on the Jaccard similarity).

Supplementary Tables

Table S3: **Number of plant species per group.**

Group	Number of species
**a**	445
**b**	149
**c**	230
**d**	299
**e**	169
**f**	277
**g**	37
**h**	180
**i**	242
**j**	136
**k**	125
**l**	95
**m**	180
**n**	180
**o**	178
**p**	186
**q**	44
**r**	59
**s**	212
**t**	274

Table S4: **Network of interactions between biogeographical regions.**

Bioregion	1	2	3	4	5	6	7	8
**1**	0.52	0.24	0.15	0.07	0	0.01	0.01	0
**2**	0.15	0.56	0.11	0.14	0.01	0.01	0.02	0
**3**	0.13	0.18	0.42	0.25	0	0	0.01	0
**4**	0.03	0.11	0.1	0.55	0.01	0.01	0.19	0.01
**5**	0.01	0.13	0	0.07	0.4	0.24	0.11	0.05
**6**	0.01	0.03	0	0.02	0.1	0.49	0.17	0.19
**7**	0	0.01	0	0.16	0.01	0.04	0.52	0.26
**8**	0	0	0	0.01	0.01	0.05	0.28	0.65

References

[1] M. G. Turner, R. H. Gardner, and R. V. O'Neill. *Landscape Ecology in Theory and Practice: Pattern and Process*. Springer-Verlag, 2001.

[2] P. J. Rousseeuw. Silhouettes: A graphical aid to the interpretation and validation of cluster analysis. *Journal of Computational and Applied Mathematics*, 20(0):53 – 65, 1987.

[3] J.B.P.A. de M. de Lamarck and A.P. Candolle. *Flore française, ou descriptions succinctes de toutes les plantes qui croissent naturellement en France, disposées selon une nouvelle méthode d'analyse, et précédées par un exposé des principes élémentaires de la botanique*. Paris, 1805.

[4] M. C. Ebach and D. F. Goujet. The first biogeographical map. *Journal of Biogeography*, 33(5):761–769, 2006.

[5] C. Flahault and M. Durand. Limite de la région méditerranéenne en France. *Publications de la Société Linnéenne de Lyon*, 5(1):9–9, 1887.

[6] P. Ozenda. *Végétation du continent Européen*. Delachaux et Niestlé, Lausanne, 1994.

[7] P. Julve. Carte phytogéographique de la france. *Cahiers de Géographie Physique*, 13:48–50, 1999.

[8] U. Bohn, G. Gollub, and C. Hettwer. *Karte der natürlichen Vegetation Europas. Bundesamt für Naturschutz*. Landwirtschaftsvlg Münster, Bonn, 1., aufl. edition, 2000.

[9] S. Rivas-Martínez, A. Penas, and T. Díaz. *Bioclimatic Map of Europe*. University of León, 2004.

[10] S. Rivas-Martínez, A. Penas, and T. Díaz. *Biogeographic Map of Europe*. University of León, 2004.

[11] European Environment Agency (EEA). *Biogeographical Regions*. Europe 2005, 2006.
